# Supplementary material for: The green algae CO2 concentrating mechanism and photorespiration jointly operate during acclimation to low CO2
Source: Nat Commun. 2025 Jun 17;16:5296. doi: 10.1038/s41467-025-60525-7 (PMC12174325; doi:10.1038/s41467-025-60525-7)
Supplement: Supplementary file 1 — Supplementary Information [file 41467_2025_60525_MOESM1_ESM.pdf]

Supplementary information for:

The green algae CO_2 concentrating mechanism and photorespiration jointly operate during acclimation to low CO_2

Ousmane Dao[1], Marie Bertrand[1], Saleh Alseekh[2,3], Florian Veillet[1], Pascaline Auroy[1], Phuong-Chi Nguyen[1], Bertrand Légeret[1], Virginie Epting[1], Amélie Morin[1], Stephan Cuiné[1], Caroline Monteil[1], Luke C.M. Mackinder[4], Adrien Burlacot[5,6], Anja Krieger-Liszkay[7], Andreas P.M. Weber[8], Alisdair R. Fernie[2,3], Gilles Peltier[1*], Yonghua Li-Beisson[1*]

[1]Aix-Marseille Université, CEA, CNRS, BIAM, UMR7265, Institut de Biosciences et Biotechnologies Aix-Marseille, CEA Cadarache, F-13115, Saint-Paul-lez-Durance, France
[2]Department of Molecular Physiology, Max Planck Institute of Molecular Plant Physiology, Potsdam-Golm, Germany
[3]Center of Plant Systems Biology and Biotechnology, 4000 Plovdiv, Bulgaria
[4]Centre for Novel Agricultural Products, Department of Biology, University of York, York YO10 5DD, UK
[5]Department of Plant Biology, Division of Biosphere Sciences and Engineering, The Carnegie Institution for Science, Stanford, CA, 94305, USA
[6]Department of Biology, Stanford University, Stanford, CA, 94305, USA
[7]Institute for Integrative Biology of the Cell (I2BC), CEA, CNRS, Université Paris-Saclay, CEDEX, 91198 Gif-sur-Yvette, France
[8]Institute of Plant Biochemistry, Cluster of Excellence on Plant Science (CEPLAS), Heinrich Heine University, 40225 Düsseldorf, Germany

[*]Correspondence to: Yonghua Li-Beisson (yonghua.li@cea.fr), Gilles Peltier (gilles.peltier@cea.fr)

ORCID ID: 0000-0002-7040-5770 (O.D.), 0000-0001-5098-1554 (M.B.), 0000-0003-2067-5235 (S.A.), 0000-0002-6892-6825 (F.V.), 0000-0002-3376-6550 (P.A.), 0000-0002-0957-4700 (B.L.), 0000-0001-6561-8432 (V.E.), 0000-0003-3318-8778 (A.M.), 0000-0002-3000-3355 (S.C.), 0000-0002-2834-6834 (C.M.), 0000-0003-1440-3233 (L.C.M.M.), 0000-0001-7434-6416 (A.B.), 0000-0001-7141-4129 (A.K.-L.), 0000-0003-0970-4672 (A.P.M.W.), 0000-0001-9000-335X (A.R.F.), 0000-0002-2226-3931 (G.P.), 0000-0003-1064-1816 (Y.L.-B.).

This PDF file includes:

Supplementary Methods
Supplementary figures 1 to 11
Supplementary References

Supplementary Methods

Genetic complementation and *Chlamydomonas* transformation. For the construction of *pPSAD::LCI20:tPSAD*, full-length genomic sequence of *LCI20* was obtained by PCR using the high fidelity KOD Hot Start DNA Polymerase (Merck Millipore) from *Chlamydomonas* genomic DNA using *LCI20* specific primers LCI20-fwd-ATG (ATGAGTGCACTTCTGGCTAG) and LCI20-rev-TAA (TTACCACCAGCCCAGCAGCT) flanked by the restriction site of *Bbs*I enzyme. Total DNA was extracted by resuspending approximately 0.5 to 1 million cells in 50 µL of NaEDTA 10 mM and heated for 10 min at 100°C in thermocycler. The PCR products were cloned under the control of the *PSAD* promoter and terminator. For transformation, the fragment harboring hygromycin resistance gene in addition to *pPSAD::LCI20:tPSAD* from the digested plasmid was incorporated into the genome of exponentially grown *Chlamydomonas* cells by electroporation and spread on TAP hygromycin (15 mg L^{-1}) agar plates. Transformants were screened by PCR using *LCI20* specific forward and reverse (TCATCGTGGTGTCGTTCTTCTTCGC and TGCGCTCTCCCAGGCCCGTCTTCTC) primers respectively. For RT-PCR, total RNA was extracted using the RNA reagent from Invitrogen (ref: 12322-012) following the same protocol as in[1]. RT-PCR was performed using LCI20-fwd-ATG and LCi20-rev-TAA primers to confirm the absence of *LCI20* transcript. *RACK1* transcript was amplified as a control gene using forward (GAGTCCAACTACGGCTACGCC) and reverse (CTCGCCAATGGTGTACTTGCAC) primers. The growth of hygromycin-resistant colonies was assessed by spot test on agar plate under VL-CO_2 conditions to screen for positive complemented lines.

***Chlamydomonas* confocal microscopy.** LCI20-Venus transgenic strain (CSI_FC1G02) harboring the pLM005-Cre06.g260450-Venus-3xFLAG construct was ordered from the *Chlamydomonas* center[2,3]. CC4533 and LCI20-Venus cells were grown mixotrophically in TAP media prior to imaging. Cells were mounted on 18-well chamber slides and overlayed with 1.5% low melting point agarose made with TP-medium. Images were obtained with a LSM880 (Zeiss) equipped with an Airyscan module using a 63× objective. Laser excitation and Emission setting for each channel used were the following: Venus (Excitation: 514 nm; Emission 525 – 500 nm) and Chlorophyll (Excitation: 633 nm; Emission 670 – 700 nm).

Generation of antibodies. Antipeptide antibodies were made by immunizing two rabbits with synthetic peptides against LCI20 (DAPSSQNGVHHDPVPEC and CDDSRKKMGSYLIQSQ), GYD1 (GEVNRILAAHQKKNKL), HPR1 (SNYAVGYNNVKVDEATKRC), GCSP (SAIARGKKPKFLVSSKC), HLA3 (RKMAEDFWSTRSAQGRNQ), LCI1 (DAEESHAMPNVHVTSDGATKV) and LCIC peptides as described[4]. Synthesis of the peptides, rabbit immunization, and purification of antibodies were performed by Proteogenix SAS (Schiltigheim). Specificities of the antibodies were then tested on total proteins extracted from whole-cell *Chlamydomonas* parental lines (CC125 and CC4533) and the *cia5* mutant.

Phylogenetic analysis. A phylogenetic tree of the dicarboxylate transporter family was built from reviewed sequences of the InterPro entry IPR030676 / CitT-rel (December 2023) and were completed with a selection of homologous sequences from the *Viridiplantae*, including *Chlorophyta*, and malate transporters from *Eubacteria*. The multiple sequence alignment was performed with MAFFT v7.49[5] using the L-INS-i strategy, and filtered for saturation with BMGE v1.12[6] using the BLOSUM62 matrix and

removing sites with > 50% gaps without entropy-based trimming, to get a final alignment containing 462 sites and 66 sequences. The tree was built using the Maximum-Likelihood (ML) algorithm with IQ-TREE[7] and the Q.LG+F+I+G4 substitution model selected by ModelFinder[8] with the Bayesian Information Criterion (BIC) and restricting model selection to the cpREV, WAG, Q.plant, Q.pfam and Q.LG matrices (**Supplementary Fig. 2**). The statistical support of the branches was estimated using a (UFBoot) approximation[9] implemented in IQ-TREE with 1000 replicates. A second tree was built from a reduced dataset of 21 sequences inspired from[10,11] (**Supplementary Fig. 1c**) using the same approach.

Supplementary Figures

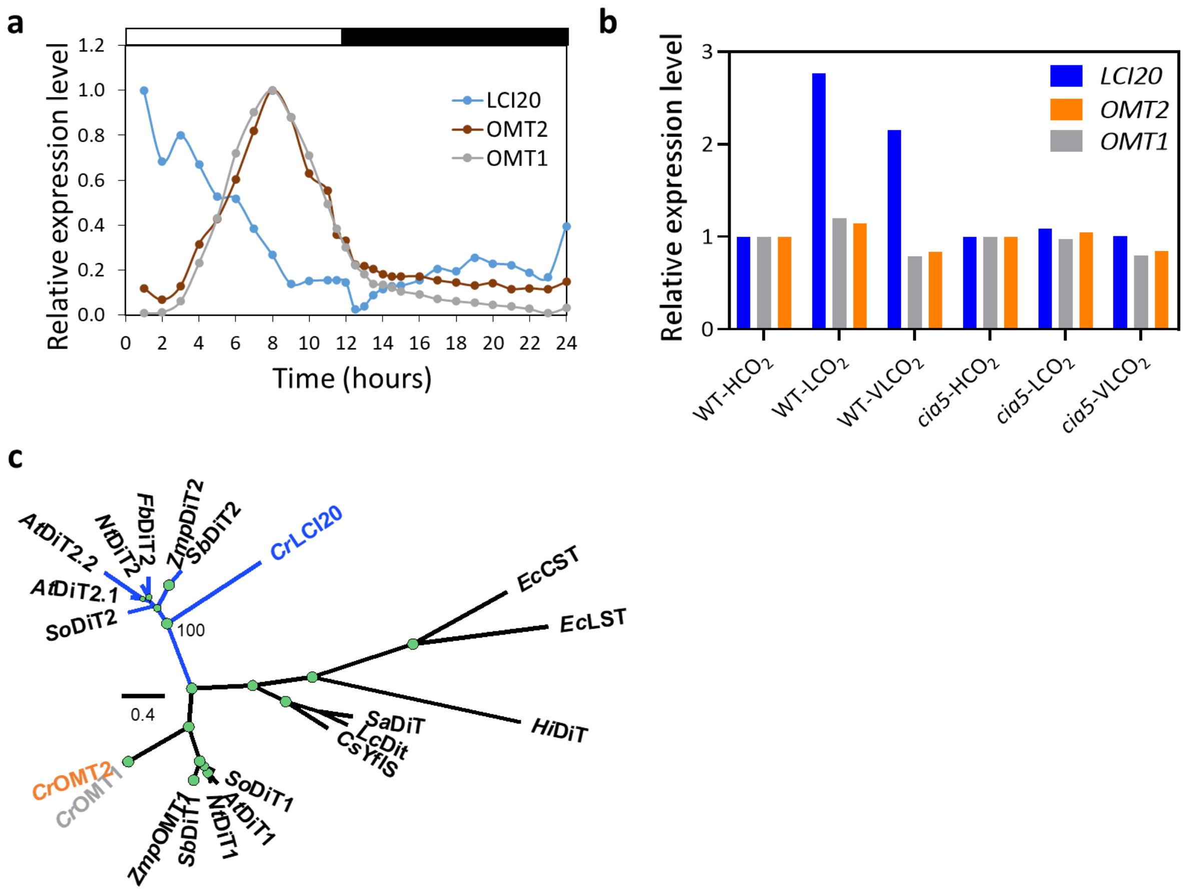

Supplementary Fig. 1. *LCI20* putatively encodes a malate transporter expressed under low CO_2 or at the onset of illumination during a day-night cycle. (**a**) Expression level of *LCI20, OMT1* and *OMT2* genes in *Chlamydomonas* WT during a day/night cycle. Data were obtained from[12]. (**b**) Expression level of *LCI20, OMT1* and *OMT2* genes in *Chlamydomonas* WT acclimated to different CO_2 levels. Data were obtained from[13]. (**c**) Unrooted Maximum Likelihood tree of the dicarboxylate transporter family in plants, *Chlamydomonas reinhardtii* and *Eubacteria* showing the affiliation of the LCI20 to the Dit2 group. The first two letters of the acronyms indicate the species (At, *Arabidopsis thaliana*; Cr, *Chlamydomonas reinhardtii*; Cs, *Clostridium saccharobutylicum*; Ec, *Escherichia coli*; Fb, *Flaveria bidentis;* Hi, *Haemophilus influenzae*; Lc, *Liquorilactobacillus cacaonum*; Nt, *Nicotiana tabacum*; Sa,

Staphylococcus aureus; So, *Spinacia oleracea*; Zm, *Zea mays*). The three following letters indicate the group of transporters (DiT, dicarboxylate transporter; TST, L-tartrate/succinate transporter; CST, citrate/succinate transporter; YlfS, 2-oxoglutarate/malate transporter). The tree was drawn to scale. The scale bar represents the number of substitutions per site. Tree topology was tested using an

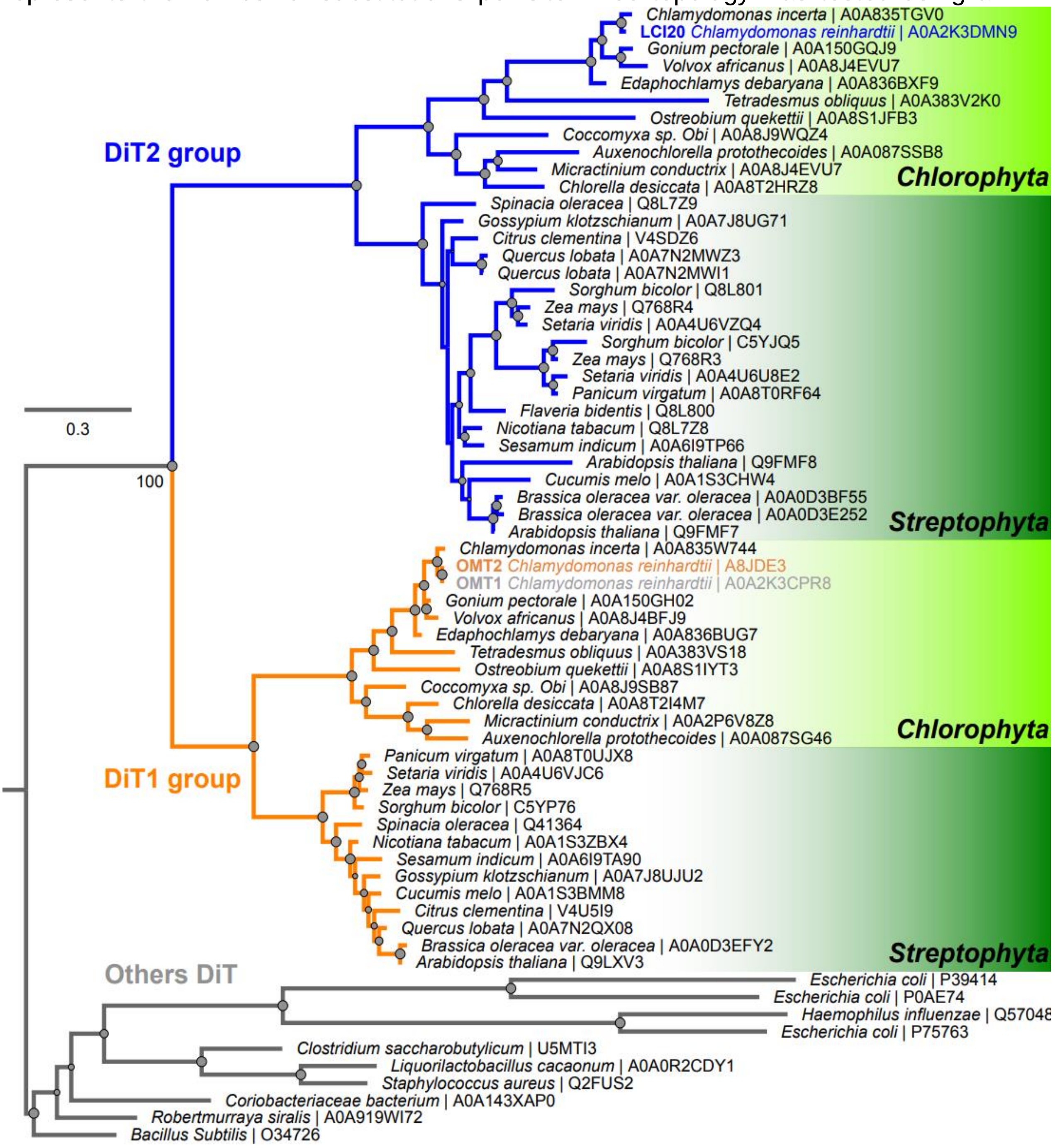

ultrafast bootstrap approximation approach with 1000 replicates. Gray circles represent bootstrap values and are drawn to scale. Dotted branches represent bacterial lineages. A full version of this tree with primary accession numbers is given in **Supplementary Fig. 2**.

Supplementary Fig. 2. Maximum likelihood tree of the dicarboxylate transporter family (Citrate carrier CitT-related / IPR030676). The tree was rooted with

Eubacteria sequences. Each sequence is associated to its primary accession number in the public database UniProtKB (https://www.uniprot.org). The tree was drawn to scale. The scale bar represents the number of substitutions per site. Tree topology was tested using an ultrafast bootstrap approximation approach with 1000 replicates. Gray circles represent bootstrap values and are drawn to scale.

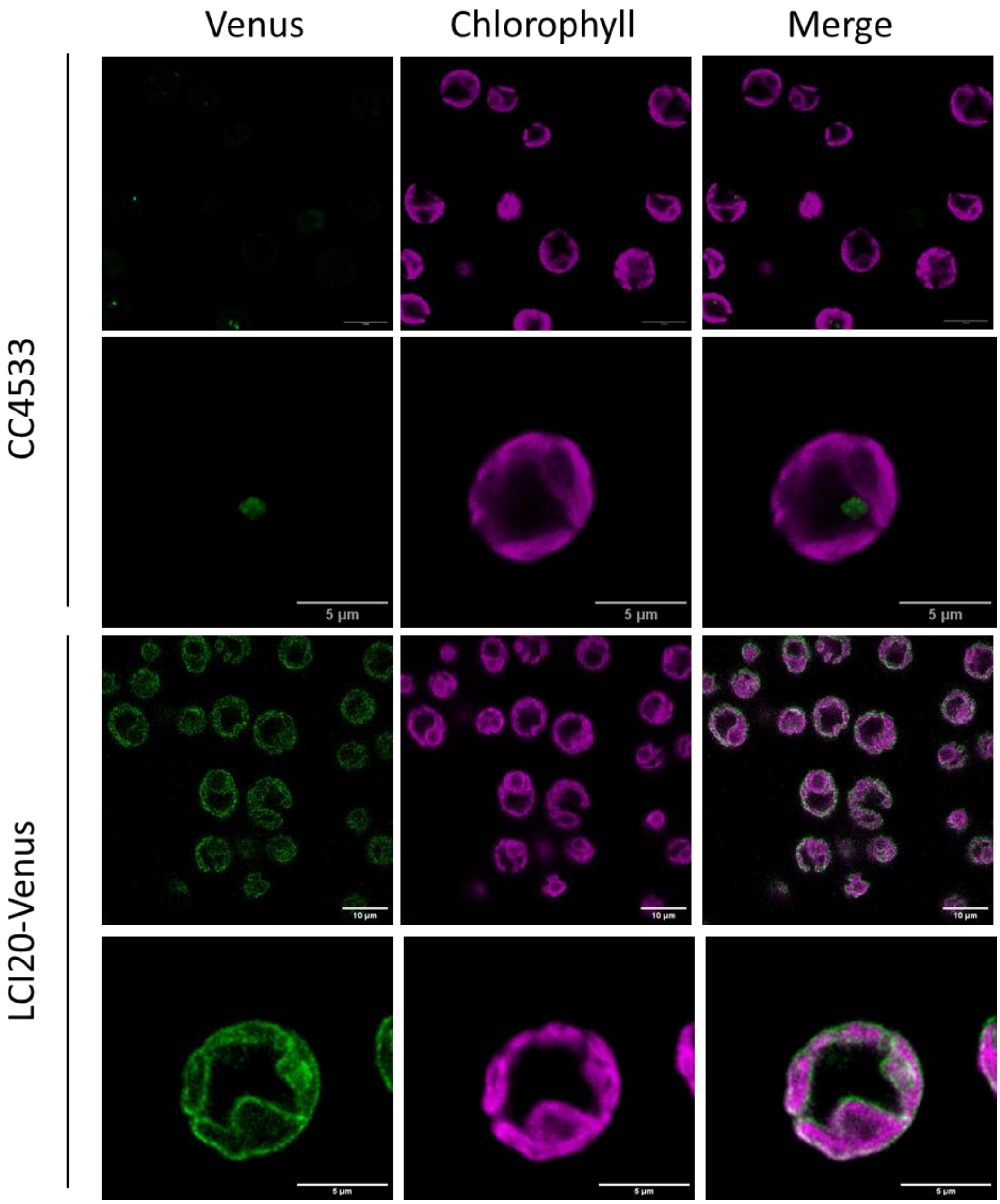

Supplementary Fig. 3. Subcellular localization of LCI20 protein fused with the Venus fluorescent reporter at its C-terminus. False colours were used to represent Venus (green) and chlorophyll (magenta) fluorescence signal. Multiple cells (top panel) and individual cell (bottom panel) are shown from CC4533 and LCI20-Venus strains. Merge represents overlayed channel of Venus and chlorophyll autofluorescence. The localization of LCI20-Venus in the chloroplast envelope was confirmed by three independent experiments. Scale bar is 5 or 10 µm as indicated.

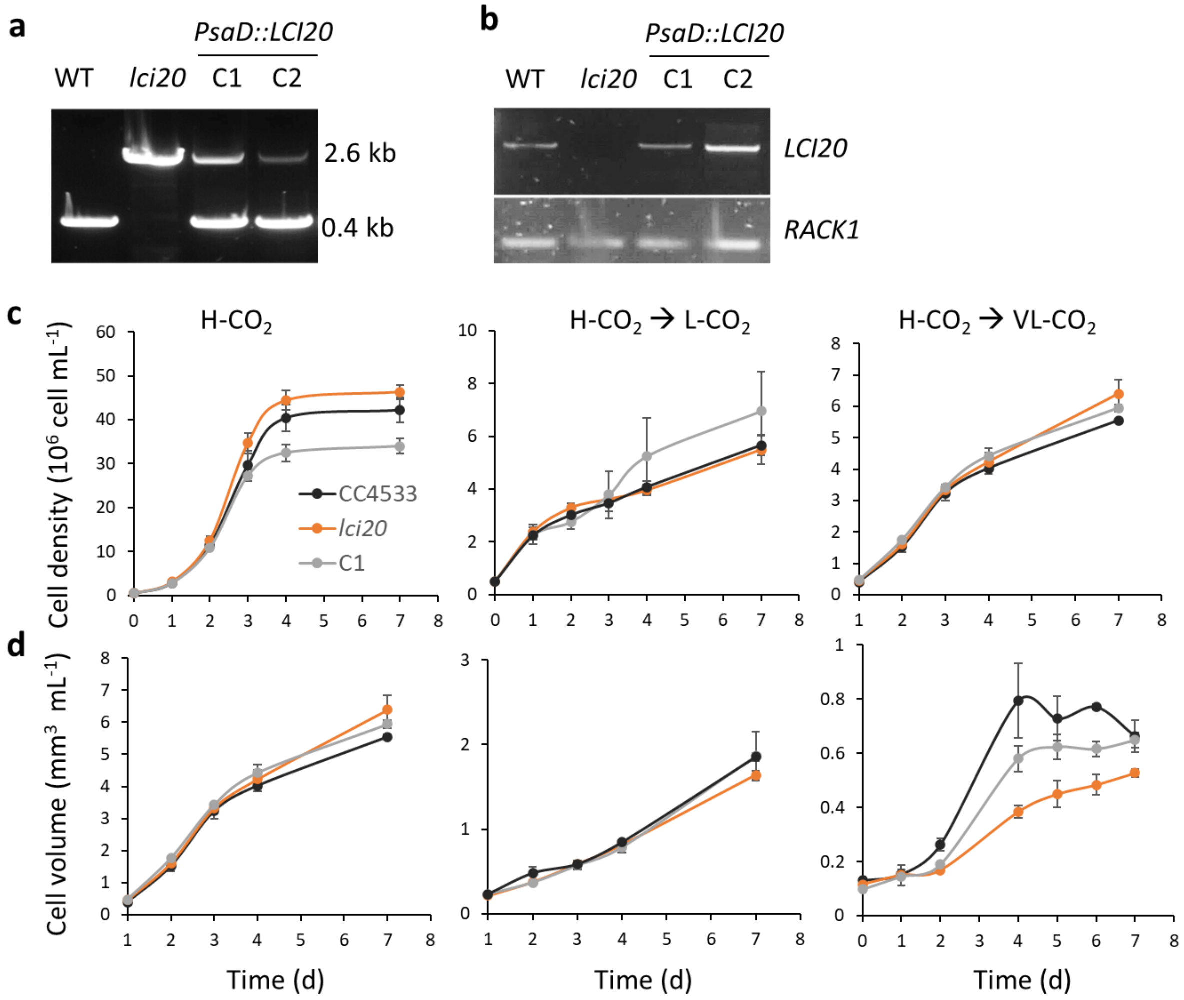

Supplementary Fig. 4. Genotyping *lci20* and of two complemented strains and growth in liquid photoautotrophic cultures under various CO_2 concentrations. (**a**) Genotyping and validation of genetic complementation of *lci20* insertional mutant by PCR using *LCI20* specific forward and reverse (TCATCGTGGTGTCGTTCTTCTTCGC and TGCGCTCTCCCAGGCCCGTCTTCTC) primers respectively. The illustration shows PCR amplification of the *LCI20* gene using primers flanking the paromomycin resistance gene insertion, resulting in a larger PCR product in *lci20* and the complemented lines showing both the endogenous *LCI20* and the transgenic full length *LCI20*. (**b**) RT-PCR showing the absence of *LCI20* transcript in the *lci20* mutant and the restauration of *LCI20* expression in the complemented lines. *RACK1* cDNA was amplified as a control gene. RT-PCR was performed using LCI20-fwd-ATG and LCi20-rev-TAA primers to confirm the absence of *LCI20* transcript. *RACK1* transcript was amplified as a control gene using forward (GAGTCCAACTACGGCTACGCC) and reverse (CTCGCCAATGGTGTACTTGCAC) primers. The results shown in (a) and (b) were repeated in three independent experiments. (**c**) Photoautotrophic growth in liquid cultures of *lci20*, its wild-type control and a complemented line following the cell number over 7 days under H-CO_2, during acclimation to L-CO_2, and VL-CO_2 21% O_2. (**d**) Same as (**c**) but growth was followed by measuring the total cellular volume. Photoautotrophic H-CO_2 grown cells were used to inoculate fresh MM growth media at 0.5 million cells mL^{-1} cell density at day zero.

Light intensity was 80 µmol photons m^{-2} s^{-1} throughout the experiment. Data are means of three biological replicates for each strain ± SD.

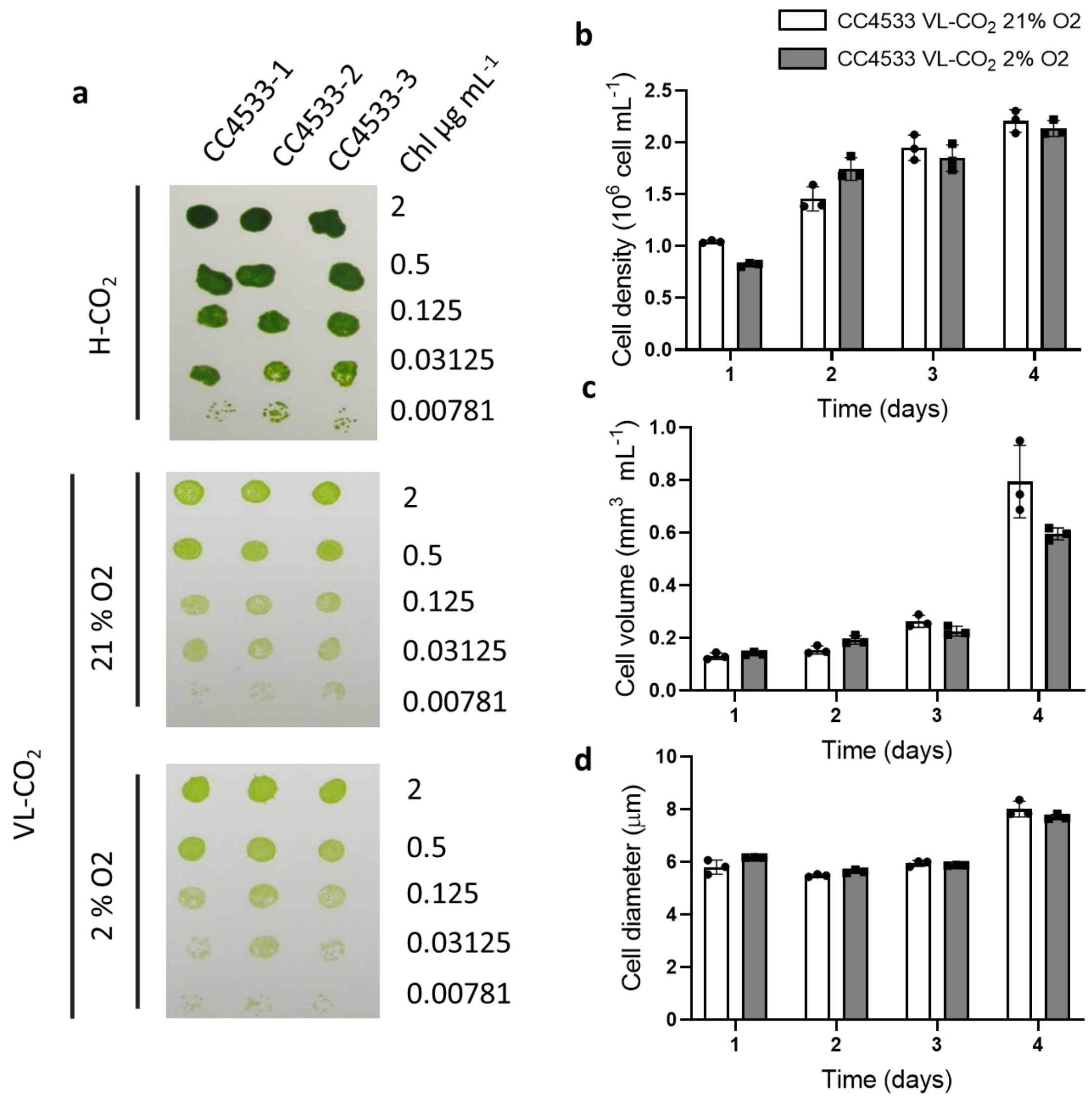

Supplementary Fig. 5. Growth characterization of Chlamydomonas wild-type CC4533 under photorespiratory and non-photorespiratory conditions. (**a**) Photoautotrophic growth of wild-type CC4533 on agar plates under H-CO_2, during acclimation to VL-CO_2 21% and 2% O_2. Images were taken after 5 days of growth under 80 µmol photons m^{-2} s^{-1}. The spot test was repeated in three independent experiments. (**b, c, d**) Photoautotrophic growth in liquid cultures of wild-type CC4533 following the cell number (b), the cell volume (c) and the cell diameter (d) during acclimation to VL-CO_2 21% and 2% O_2. Photoautotrophic H-CO_2 grown cells were used for spot tests (**a**) or inoculating fresh MM growth media at 10^6 cells mL^{-1} cell density at day zero (**b-d**). Light intensity was 80 µmol photons m^{-2} s^{-1} throughout the experiments. Data are means of three biological replicates for each strain +/- SD.

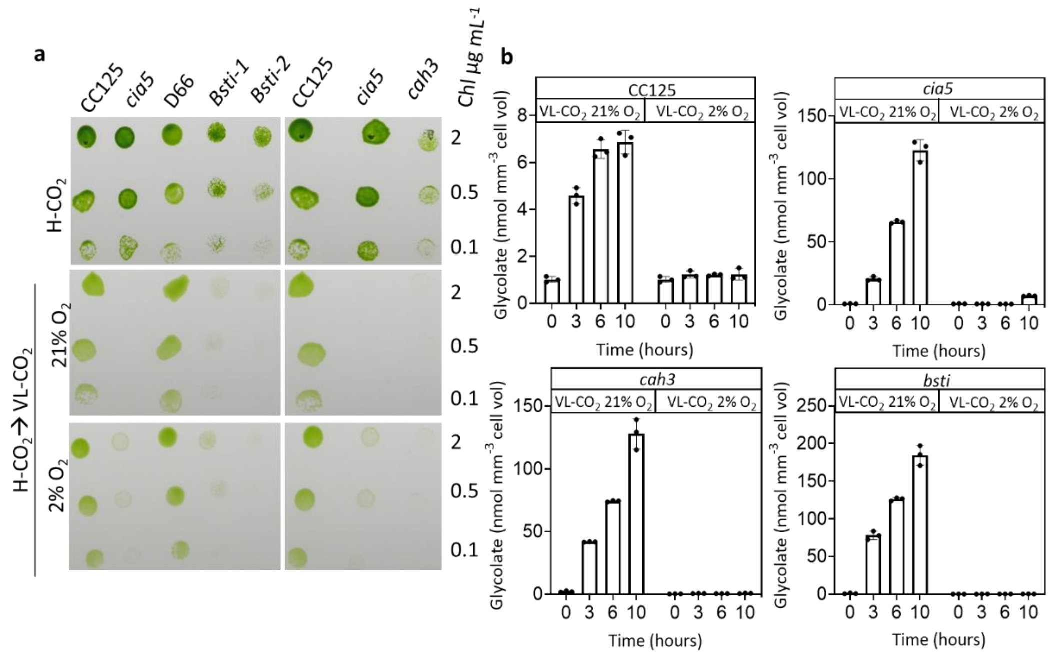

Supplementary Fig. 6. Characterization of low CO_2 inducible bestrophins (*bsti*) and thylakoid lumen carbonic anhydrase (*cah3*) deficient mutants under photorespiratory and non-photorespiratory conditions. (**a**) Photoautotrophic growth of *bsti* and *cah3* mutants on agar plates exposed to various CO_2 and O_2 concentrations. The spot test was repeated in three independent experiments. Cells were grown in liquid culture in flasks photo-autotrophically under H-CO_2 prior to spot test. Images were taken after 3 days (H-CO_2) or 5 days (VL-CO_2) of growth under 80 µmol photons m^{-2} s^{-1}. (**b**) Quantification of the glycolate concentration in the culture medium after a transition from H-CO_2 to VL-CO_2 at 21% or 2% O_2. Bars show the average and dots show data from independent biological replicates (n=3 ± SD).

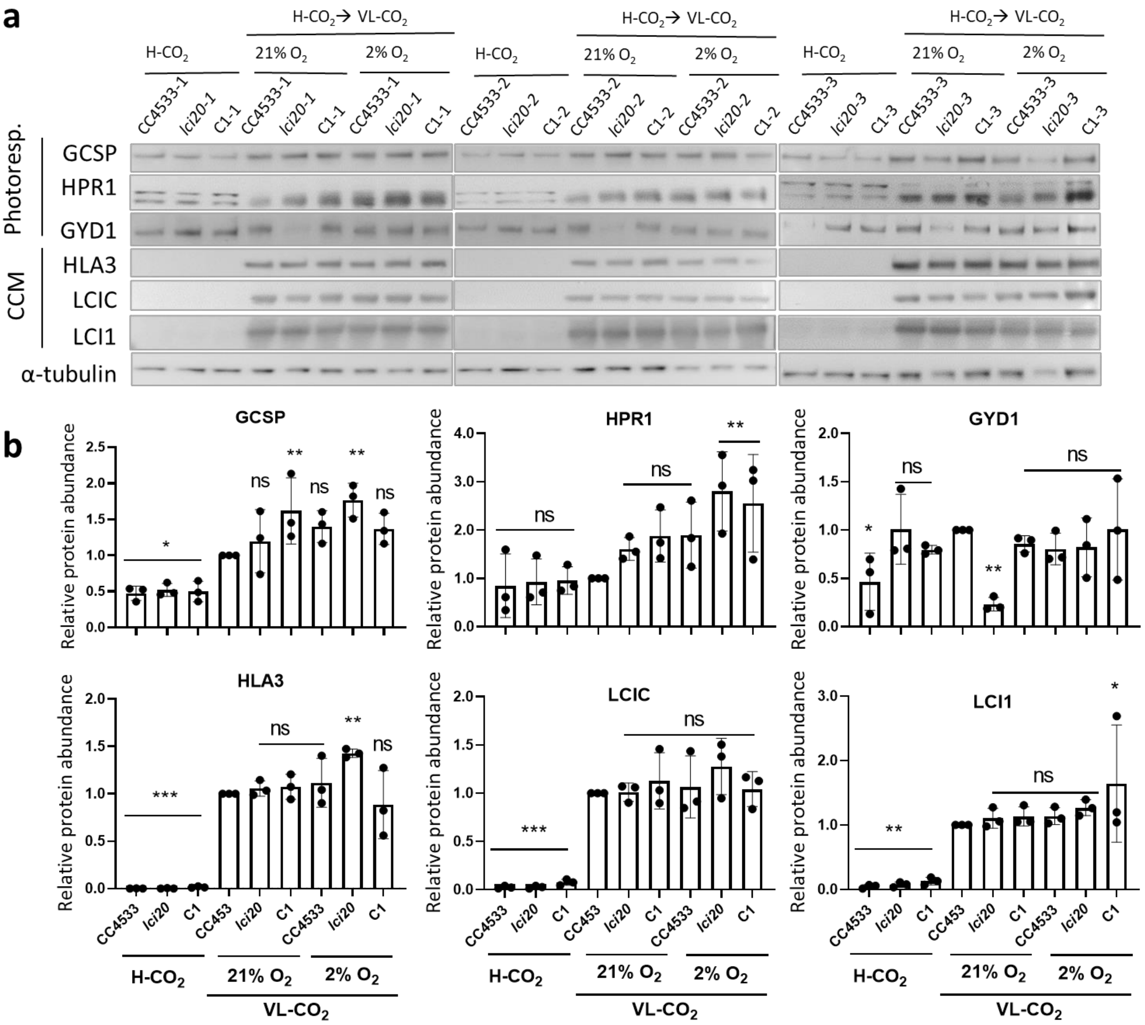

Supplementary Fig. 7: Quantification of photorespiration and CCM related proteins under H-CO_2 and during acclimation to VL-CO_2 21% O_2 or 2% O_2 in *lci20*. (a) Immunoblot analysis of representative CCM and photorespiration related proteins from three biological replicates of *lci20*, its wild-type control CC4533 and one complemented line (C1). a-tubulin was used as a loading control. (b) Relative abundance of CCM and photorespiratory related proteins in *lci20,* its wild-type control CC4533 and one complemented line using the immunoblots shown in (a). All the immunoblots were normalized by α-tubulin signals. Data are means of three independent biological replicates (n=3 ± SD). Asterisks represent statistically significant difference compared to the wild-type CC4533 under VL-CO_2 21% O_2 (* $p \leq 0.05$, ** $p \leq 0.01$, *** $p \leq 0.001$ and **** $p \leq 0.0001$) using one-way ANOVA. Cells were cultivated photo-autotrophically under H-CO_2 and 80 µmol photons m^{-2} s^{-1} and then acclimated for 20 h at the indicated CO_2 and O_2 levels prior to immunoblot analysis. Uncropped immunoblots are provided as a Source Data file.

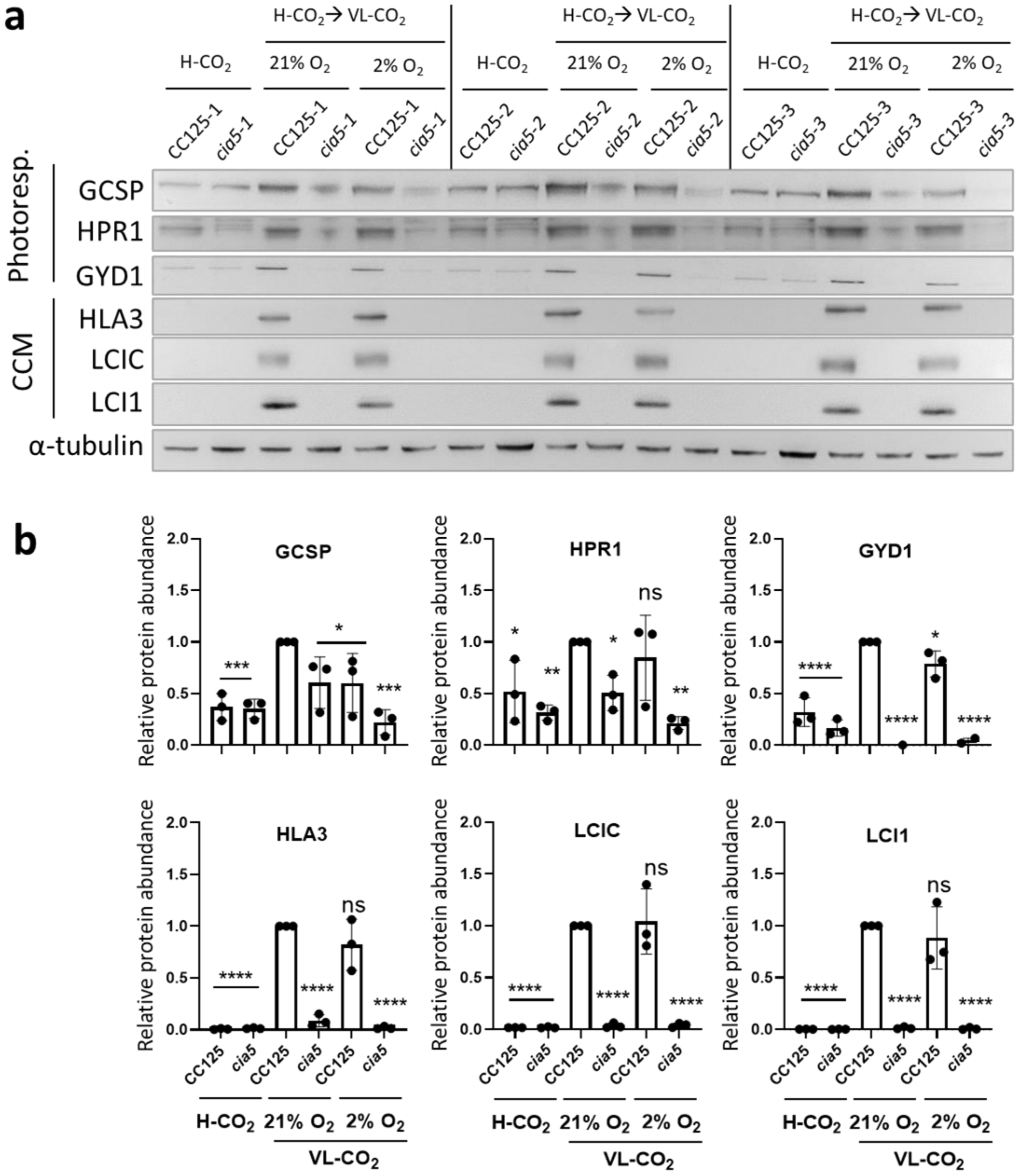

Supplementary Fig. 8: Quantification of photorespiration and CCM related proteins under H-CO_2 and during acclimation to VL-CO_2 21% O_2 or 2% O_2 in *cia5*. (a) Immunoblot analysis of representative CCM and photorespiration related proteins from three biological replicates of *cia5* and its wild-type control CC125. a-tubulin was used as a loading control. (b) Relative abundance of CCM and photorespiratory related proteins in *cia5* and its wild-type control CC125 using the immunoblots shown in (a). All the immunoblots were normalized by α-tubulin signals. Data are means of three independent biological replicates (n=3 ± SD). Asterisks represent statistically significant difference compared to the wild-type CC125 under VL-CO_2 21% O_2 (* $p \leq 0.05$, ** $p \leq 0.01$, *** $p \leq 0.001$ and **** $p \leq 0.0001$) using one-way ANOVA. Cells were cultivated photo-autotrophically under H-CO_2 and 80 μmol photons m^{-2} s^{-1} and then acclimated for 20 h at the indicated CO_2 and O_2 levels prior to immunoblot analysis. Uncropped immunoblots are provided as a Source Data file.

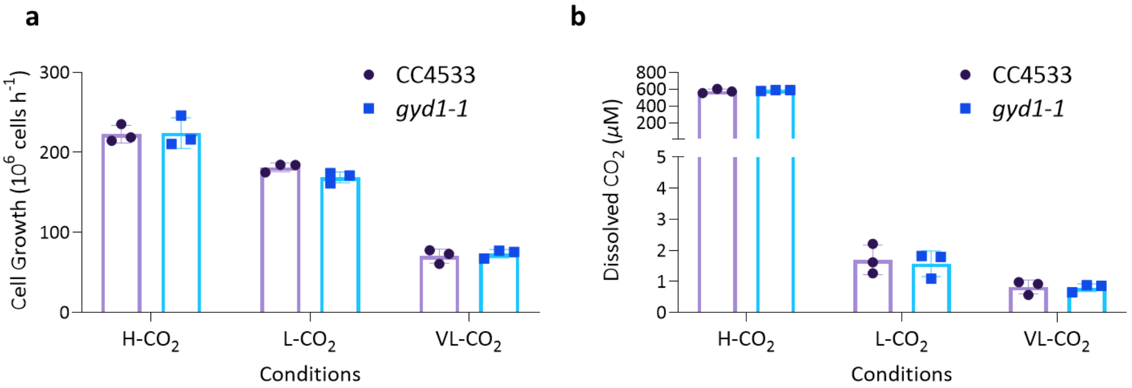

Supplementary Fig. 9. Growth characterization of glycolate dehydrogenase *gyd1-1* mutant under various CO_2 concentrations. The *gyd1-1* mutant and its wild-type control (CC4533) were grown photoautotrophically in 1L photobioreactors operated as turbidostats under a light intensity of 125 µmol photons. m^{-2} s^{-1} in the presence of 4% CO_2 (H-CO_2), 1000 ppm (L-CO_2) and 400 ppm (VL-CO_2) in air. (a) Growth was determined from dilution rates based on cell counting. (b) Dissolved CO_2 concentration present in photobioreactors was determined by membrane inlet mass spectrometry upon rapid filtration to ensure that L-CO_2 and VL-CO_2 have been reached (note that the dissolved CO_2 concentration in equilibrium with air is around 10 µM).

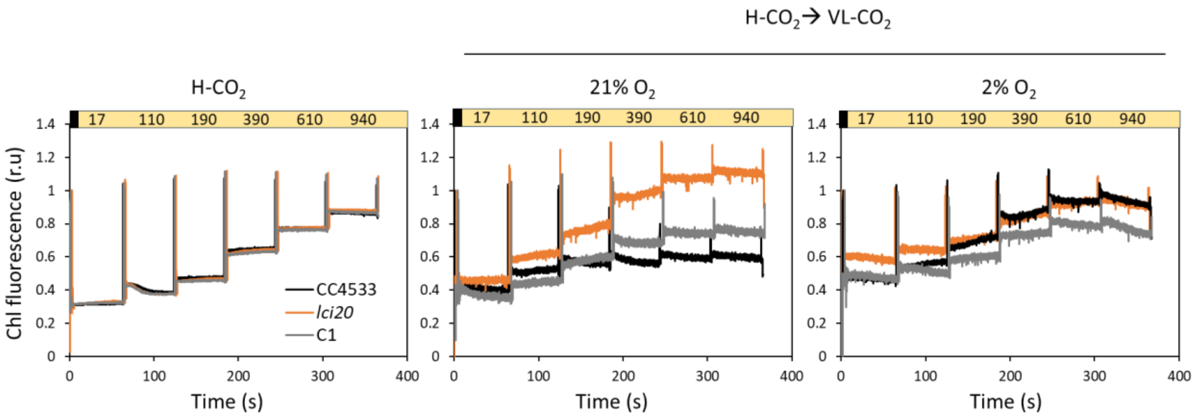

Supplementary Fig. 10. Representative chlorophyll fluorescence traces of *lci20*, its wild-type control and one complemented line recorded under various CO_2 levels. Chlorophyll fluorescence measurements were carried out using actinic red light of stepwise increasing intensity: 0, 17, 110, 190, 390, 610 and 940 µmol photons m^{-2} s^{-1} respectively. The black and yellow boxes represent the dark and light phases respectively. Saturating flashes, indicated by vertical lines, were supplied every 60s. Data are normalized on initial F*m* measurements and traces are shifted few seconds to allow clarity. The initial F*m* and F_0 was determined after a 15 min dark adaptation. Chlorophyll fluorescence traces such as the ones shown here were used to calculate data shown in Fig. 4 as described in methods section.

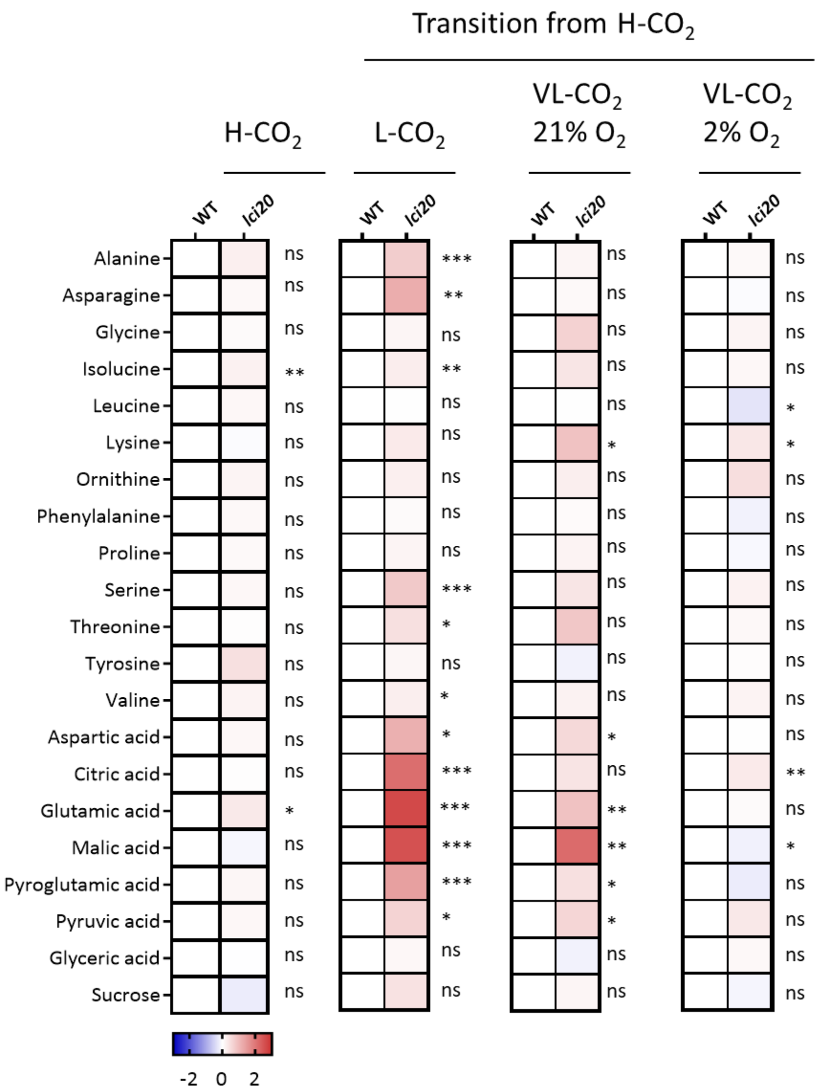

Supplementary Fig. 11. Metabolomics analysis of *lci20* and its wild-type control acclimated to H-CO_2 and during acclimation to L- or VL-CO_2 conditions. Heatmap shows fold-changes of metabolites in the *lci20* as compared to the WT under H-CO_2, and during acclimation to L-CO_2, VL-CO_2 21% O_2 and VL-CO_2 2% O_2 for 20 h. Shown represents the average of eight biological replicates for each strain. Asterisks represent statistically significant differences compared to WT CC4533 (* $p \leq 0.05$, ** $p \leq 0.01$ and *** $p \leq 0.001$) using two-way ANOVA.

Supplementary References

1. Nguyen, H. M. *et al.* The Green Microalga Chlamydomonas reinhardtii Has a Single ω-3 Fatty Acid Desaturase That Localizes to the Chloroplast and Impacts Both Plastidic and Extraplastidic Membrane Lipids. *Plant Physiology* **163**, 914–928 (2013).
2. Mackinder, L. C. M. *et al.* A Spatial Interactome Reveals the Protein Organization of the Algal CO2-Concentrating Mechanism. *Cell* **171**, 133-147.e14 (2017).
3. Wang, L. *et al.* A chloroplast protein atlas reveals punctate structures and spatial organization of biosynthetic pathways. *Cell* **186**, 3499-3518.e14 (2023).

4. Yamano, T. *et al.* Light and Low-CO2-Dependent LCIB–LCIC Complex Localization in the Chloroplast Supports the Carbon-Concentrating Mechanism in Chlamydomonas reinhardtii. *Plant Cell Physiol* **51**, 1453–1468 (2010).
5. Katoh, K. & Standley, D. M. MAFFT multiple sequence alignment software version 7: improvements in performance and usability. *Mol Biol Evol* **30**, 772–780 (2013).
6. Criscuolo, A. & Gribaldo, S. BMGE (Block Mapping and Gathering with Entropy): a new software for selection of phylogenetic informative regions from multiple sequence alignments. *BMC Evol Biol* **10**, 210 (2010).
7. Minh, B. Q. *et al.* IQ-TREE 2: New Models and Efficient Methods for Phylogenetic Inference in the Genomic Era. *Molecular Biology and Evolution* **37**, 1530–1534 (2020).
8. Kalyaanamoorthy, S., Minh, B. Q., Wong, T. K. F., von Haeseler, A. & Jermiin, L. S. ModelFinder: fast model selection for accurate phylogenetic estimates. *Nat Methods* **14**, 587–589 (2017).
9. Hoang, D. T., Chernomor, O., von Haeseler, A., Minh, B. Q. & Vinh, L. S. UFBoot2: Improving the Ultrafast Bootstrap Approximation. *Molecular Biology and Evolution* **35**, 518–522 (2018).
10. Weber, A. & Flügge, U. Interaction of cytosolic and plastidic nitrogen metabolism in plants. *Journal of Experimental Botany* **53**, 865–874 (2002).
11. Renné, P. *et al.* The Arabidopsis mutant dct is deficient in the plastidic glutamate/malate translocator DiT2. *The Plant Journal* **35**, 316–331 (2003).
12. Zones, J. M., Blaby, I. K., Merchant, S. S. & Umen, J. G. High-Resolution Profiling of a Synchronized Diurnal Transcriptome from Chlamydomonas reinhardtii Reveals Continuous Cell and Metabolic Differentiation. *Plant Cell* **27**, 2743–2769 (2015).
13. Fang, W. *et al.* Transcriptome-Wide Changes in Chlamydomonas reinhardtii Gene Expression Regulated by Carbon Dioxide and the CO2-Concentrating Mechanism Regulator CIA5/CCM1[W][OA]. *Plant Cell* **24**, 1876–1893 (2012).
