# Supplementary material for: The green algae CO2 concentrating mechanism and photorespiration jointly operate during acclimation to low CO2
Source: Nat Commun. 2025 Jun 17;16:5296. doi: 10.1038/s41467-025-60525-7 (PMC12174325; doi:10.1038/s41467-025-60525-7)
Supplement: Supplementary file 2 — Description of Additional Supplementary Information [file 41467_2025_60525_MOESM2_ESM.docx]

**Description of Additional Supplementary Files**

File Name: Supplementary Data 1

Description: List of metabolites analysed by GC-MS. Data are normalized by cell number and correspond to the heatmaps shown in supplementary figure 11.
