# Supplementary material for: The green algae CO2 concentrating mechanism and photorespiration jointly operate during acclimation to low CO2
Source: Nat Commun. 2025 Jun 17;16:5296. doi: 10.1038/s41467-025-60525-7 (PMC12174325; doi:10.1038/s41467-025-60525-7)
Supplement: Supplementary file 4 — Reporting Summary [file 41467_2025_60525_MOESM4_ESM.pdf]

nature portfolio

Corresponding author(s): Yonghua Li-Beisson

Last updated by author(s): May 21, 2025

Reporting Summary

Nature Portfolio wishes to improve the reproducibility of the work that we publish. This form provides structure for consistency and transparency in reporting. For further information on Nature Portfolio policies, see our Editorial Policies and the Editorial Policy Checklist.

Statistics

For all statistical analyses, confirm that the following items are present in the figure legend, table legend, main text, or Methods section.

n/a	Confirmed	
☐	☒	The exact sample size (n) for each experimental group/condition, given as a discrete number and unit of measurement
☐	☒	A statement on whether measurements were taken from distinct samples or whether the same sample was measured repeatedly
☐	☒	The statistical test(s) used AND whether they are one- or two-sided<br>*Only common tests should be described solely by name; describe more complex techniques in the Methods section.*
☒	☐	A description of all covariates tested
☒	☐	A description of any assumptions or corrections, such as tests of normality and adjustment for multiple comparisons
☐	☒	A full description of the statistical parameters including central tendency (e.g. means) or other basic estimates (e.g. regression coefficient) AND variation (e.g. standard deviation) or associated estimates of uncertainty (e.g. confidence intervals)
☐	☒	For null hypothesis testing, the test statistic (e.g. F, t, r) with confidence intervals, effect sizes, degrees of freedom and P value noted<br>*Give P values as exact values whenever suitable.*
☒	☐	For Bayesian analysis, information on the choice of priors and Markov chain Monte Carlo settings
☒	☐	For hierarchical and complex designs, identification of the appropriate level for tests and full reporting of outcomes
☒	☐	Estimates of effect sizes (e.g. Cohen's d, Pearson's r), indicating how they were calculated

Our web collection on statistics for biologists contains articles on many of the points above.

Software and code

Policy information about availability of computer code

Data collection	*Provide a description of all commercial, open source and custom code used to collect the data in this study, specifying the version used OR state that no software was used.*
Data analysis	*Provide a description of all commercial, open source and custom code used to analyse the data in this study, specifying the version used OR state that no software was used.*

For manuscripts utilizing custom algorithms or software that are central to the research but not yet described in published literature, software must be made available to editors and reviewers. We strongly encourage code deposition in a community repository (e.g. GitHub). See the Nature Portfolio guidelines for submitting code & software for further information.

Data

Policy information about availability of data

All manuscripts must include a data availability statement. This statement should provide the following information, where applicable:

- Accession codes, unique identifiers, or web links for publicly available datasets
- A description of any restrictions on data availability
- For clinical datasets or third party data, please ensure that the statement adheres to our policy

Genes studied in this article can be found on Phytozome (v6.1 genome: https://phytozome-next. jgi.doe.gov/) under the loci Cre06.g260450 (LCI20), Cre06.g295450 (HPR1), Cre06.g288700 (GYD1), Cre12.g534800 (GCSP), Cre03.g162800 (LCI1), Cre02.g097800 (HLA3), Cre06.g307500 (LCIC) and Cre02.g096300 (CIA5). All antibodies used are available upon request to corresponding authors. All source data are provided with this paper as a Source Data File.

Research involving human participants, their data, or biological material

Policy information about studies with human participants or human data. See also policy information about sex, gender (identity/presentation), and sexual orientation and race, ethnicity and racism.

Reporting on sex and gender	*Use the terms sex (biological attribute) and gender (shaped by social and cultural circumstances) carefully in order to avoid confusing both terms. Indicate if findings apply to only one sex or gender; describe whether sex and gender were considered in study design; whether sex and/or gender was determined based on self-reporting or assigned and methods used.*<br>*Provide in the source data disaggregated sex and gender data, where this information has been collected, and if consent has been obtained for sharing of individual-level data; provide overall numbers in this Reporting Summary. Please state if this information has not been collected.*<br>*Report sex- and gender-based analyses where performed, justify reasons for lack of sex- and gender-based analysis.*
Reporting on race, ethnicity, or other socially relevant groupings	*Please specify the socially constructed or socially relevant categorization variable(s) used in your manuscript and explain why they were used. Please note that such variables should not be used as proxies for other socially constructed/relevant variables (for example, race or ethnicity should not be used as a proxy for socioeconomic status).*<br>*Provide clear definitions of the relevant terms used, how they were provided (by the participants/respondents, the researchers, or third parties), and the method(s) used to classify people into the different categories (e.g. self-report, census or administrative data, social media data, etc.)*<br>*Please provide details about how you controlled for confounding variables in your analyses.*
Population characteristics	*Describe the covariate-relevant population characteristics of the human research participants (e.g. age, genotypic information, past and current diagnosis and treatment categories). If you filled out the behavioural & social sciences study design questions and have nothing to add here, write "See above."*
Recruitment	*Describe how participants were recruited. Outline any potential self-selection bias or other biases that may be present and how these are likely to impact results.*
Ethics oversight	*Identify the organization(s) that approved the study protocol.*

Note that full information on the approval of the study protocol must also be provided in the manuscript.

Field-specific reporting

Please select the one below that is the best fit for your research. If you are not sure, read the appropriate sections before making your selection.

☒ Life sciences ☐ Behavioural & social sciences ☐ Ecological, evolutionary & environmental sciences

For a reference copy of the document with all sections, see nature.com/documents/nr-reporting-summary-flat.pdf

Life sciences study design

All studies must disclose on these points even when the disclosure is negative.

Sample size	Three biologically independent cultures were used for most of the experiments except for metabolites analysis where eight independent biological cultures were used.
Data exclusions	No data were excluded from this study
Replication	Experiments were reproduced several times to ensure the consistency of the phenotype.
Randomization	In this study, mutants impaired in genes of interest were compared to their respective controls (so called wild-type) and complemented lines when necessary.
Blinding	Blinding was not relevant to this study. We used known mutants impaired in genes of interest.

Reporting for specific materials, systems and methods

We require information from authors about some types of materials, experimental systems and methods used in many studies. Here, indicate whether each material, system or method listed is relevant to your study. If you are not sure if a list item applies to your research, read the appropriate section before selecting a response.

Materials & experimental systems

n/a	Involved in the study
☐	☒ Antibodies
☐	☒ Eukaryotic cell lines
☒	☐ Palaeontology and archaeology
☒	☐ Animals and other organisms
☒	☐ Clinical data
☒	☐ Dual use research of concern
☐	☒ Plants

Methods

n/a	Involved in the study
☒	☐ ChIP-seq
☒	☐ Flow cytometry
☒	☐ MRI-based neuroimaging

Antibodies

Antibodies used	Antipeptide antibodies were made by immunizing two rabbits with synthetic peptides against LCI20 (DAPSSQNGVHHDPVPEC and CDDSRKKMGSYLIQSQ), GYD1 (GEVNRILAAHQKKNKL), HPR1 (SNYAVGYNNVKVDEATKRC), GCSP (SAIARGKKPKFLVSSKC), HLA3 (RKMAEDFWSTRSAQGRNQ), LCI1 (DAEESHAMPNVHVTSDGATKV) and LCIC peptides as described3. Synthesis of the peptides, rabbit immunization, and purification of antibodies were performed by Proteogenix SAS (Schiltigheim).
Validation	After the ELISA test by the company, we have tested the specificities of the antibodies on total proteins extracted from whole-cell Chlamydomonas parental lines (CC125 and CC4533) and the cia5 mutant.

Eukaryotic cell lines

Policy information about cell lines and Sex and Gender in Research

Cell line source(s)	All cells (including mutants) were purchased from the Chlamydomonas resource center
Authentication	Mutants were authenticated by checking the absence of the gene product (i.e. proteins) using immunoblot and also by checking the insertion of the cassette in the genes.
Mycoplasma contamination	Not tested
Commonly misidentified lines (See ICLAC register)	Did not apply in this study

Plants

Seed stocks	All cells (including mutants) were purchased from the Chlamydomonas resource center
Novel plant genotypes	No mutants were produced in this study. We have only performed complementation of lci20 mutants by expressing the mutant the native endogenous gene under PSAD promoter.
Authentication	The complementated lines were validated by immunoblots showing the rescue of gene expression.
